# A bibliometric analysis of the neurotoxicity of anesthesia in the developing brain from 2002 to 2021

**DOI:** 10.3389/fneur.2023.1185900

**Published:** 2023-04-27

**Authors:** Ying Cao, Yang Sun, Xiao Liu, Kang Yu, Danyang Gao, Jiaojiao Yang, Huihui Miao, Tianzuo Li

**Affiliations:** Department of Anesthesiology, Beijing Shijitan Hospital, Capital Medical University, Beijing, China

**Keywords:** neurotoxicity, anesthesia, developing brain, bibliometric analysis, hotspots

## Abstract

**Background:**

The neurotoxicity effects of anesthetic exposure on the developing brain have been one of the current research hotspots and numerous articles were published in the past decades. However, the quality and comparative information of these articles have not been reported. This research aimed to provide a comprehensive overview of the current state of the field by investigating research hotspots and publication trends concerning the neurotoxicity of anesthesia in the developing brain.

**Materials and methods:**

On 15 June 2022, we searched articles on the neurotoxicity of anesthesia in the developing brain through the Science Citation Index databases from 2002 to 2021. Data of the author, title, publication, funding agency, date of publication, abstract, type of literature, country, journal, keywords, number of citations, and research direction were collected for further analysis.

**Results:**

We searched and analyzed 414 articles in English on the field of neurotoxicity of anesthesia in the developing brain from 2002 to 2021. The country with the largest number of publications was The United States (US) (*n* = 226), which also had the largest total number of citations (10,419). Research in this field reached a small peak in 2017. Furthermore, the largest number of articles were published in three journals, Anesthesiology, Anesthesia and Analgesia, and Pediatric Anesthesia. The top 20 articles that were cited most often were studied. In addition, the top hotspots of this area in clinical investigations and basic research were analyzed separately.

**Conclusion:**

This study provided an overview of the development in the neurotoxicity of anesthesia in the developing brain using bibliometric analysis. Current clinical studies in this area were mainly retrospective; in the future, we should place more emphasis on prospective, multicenter, long-term monitoring clinical studies. More basic research was also needed on the mechanisms of neurotoxicity of anesthesia in the developing brain.

## Introduction

Advances in medical technology have led to a greater number of babies receiving surgery under anesthesia at an early stage after birth. It remains unclear whether anesthesia adversely affects the child's ability to learn and its cognitive function later in life (1).Some fundamental studies have confirmed that anesthesia is harmful to neurons in the developing brain and causes cognitive damage. However, other reports implied that anesthetic exposure is beneficial to brain development (2–4). Many retrospective clinical studies have shown that receiving anesthesia in childhood increases the risk of later studying, awareness, and behavioral abnormalities (5–7). Several studies also demonstrated that receiving anesthesia at an early age had no appreciable impact on neurodevelopment (8).

Although the neurotoxicity of anesthesia in infants and children has received widespread attention in the field of anesthesia, various scientific questions related to the neurotoxicity of anesthesia remain unanswered and require continued research. Therefore, a detailed assessment of the neurotoxicity of anesthesia in the developing brain is necessary to better prepare for further studies.

Bibliometric analysis is the quantitative analysis of the literature on a particular topic using mathematical and statistical methods, preferring to quantify the comprehensive body of knowledge on the topic studied and to guide future research directions by highlighting the latest research trends (9–11). However, there are no relevant bibliometric articles that summarized the latest progress in this field. Our study aimed to provide a thorough overview of the state of the field by examining research hotspots and publication trends related to the neurotoxicity of anesthesia in the developing brain.

## Materials and methods

### Search strategy

**Figure 1:**
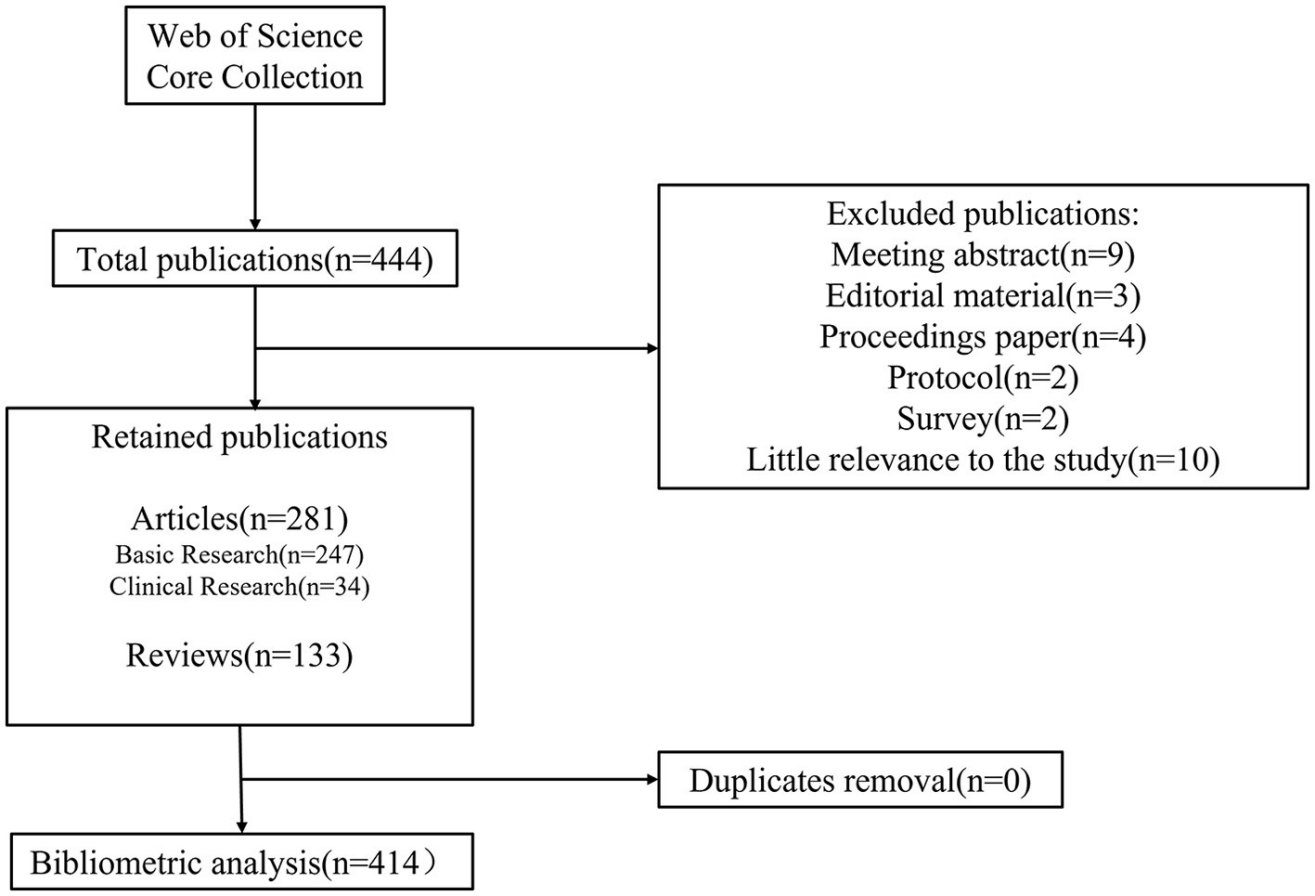
Flow chart for literature screening.

We conducted an online literature search using the Science Citation Index databases on 15 June 2022. To reduce the data errors caused by frequent database updates, we completed the search of data in one go. The search strategy was topic = (anesthes^*^ neurotoxic^*^) and (developing brain^*^); the publication date = (2002-01-01) to (2022-06-15). We only collected literature in English and limited the article type to “article or review”. Figure 1 illustrates the process of collecting literature. A total of 444 articles emerged from our search strategy, and we excluded Meeting abstract (*n* = 9), Editorial material (*n* = 3), Proceedings paper (*n* = 4), Protocol (*n* = 2), Survey (*n* = 2), and 10 articles with little relevance to the study. We ended up with 133 reviews and 281 original articles, which consisted of 247 basic studies and 34 clinical studies. We collected the following bibliometric information: author, title, publication, funding agency, date of publication, abstract, type of literature, country, journal, keywords, number of citations, and research direction. No other exclusion criteria were used.

### Statistical analysis

To carry out a bibliometric study and create graphs, we used CiteSpace software. GraphPad Prism 8.0 (GraphPad, San Diego, CA, USA) was used to conduct the statistical analysis. Numbers or percentages were used to represent data. The two-tailed Pearson correlation method was used to conduct correlation analysis. Statistical significance was set at *P* < 0.05.

## Results

### Year and country of publication

**Figure 2:**
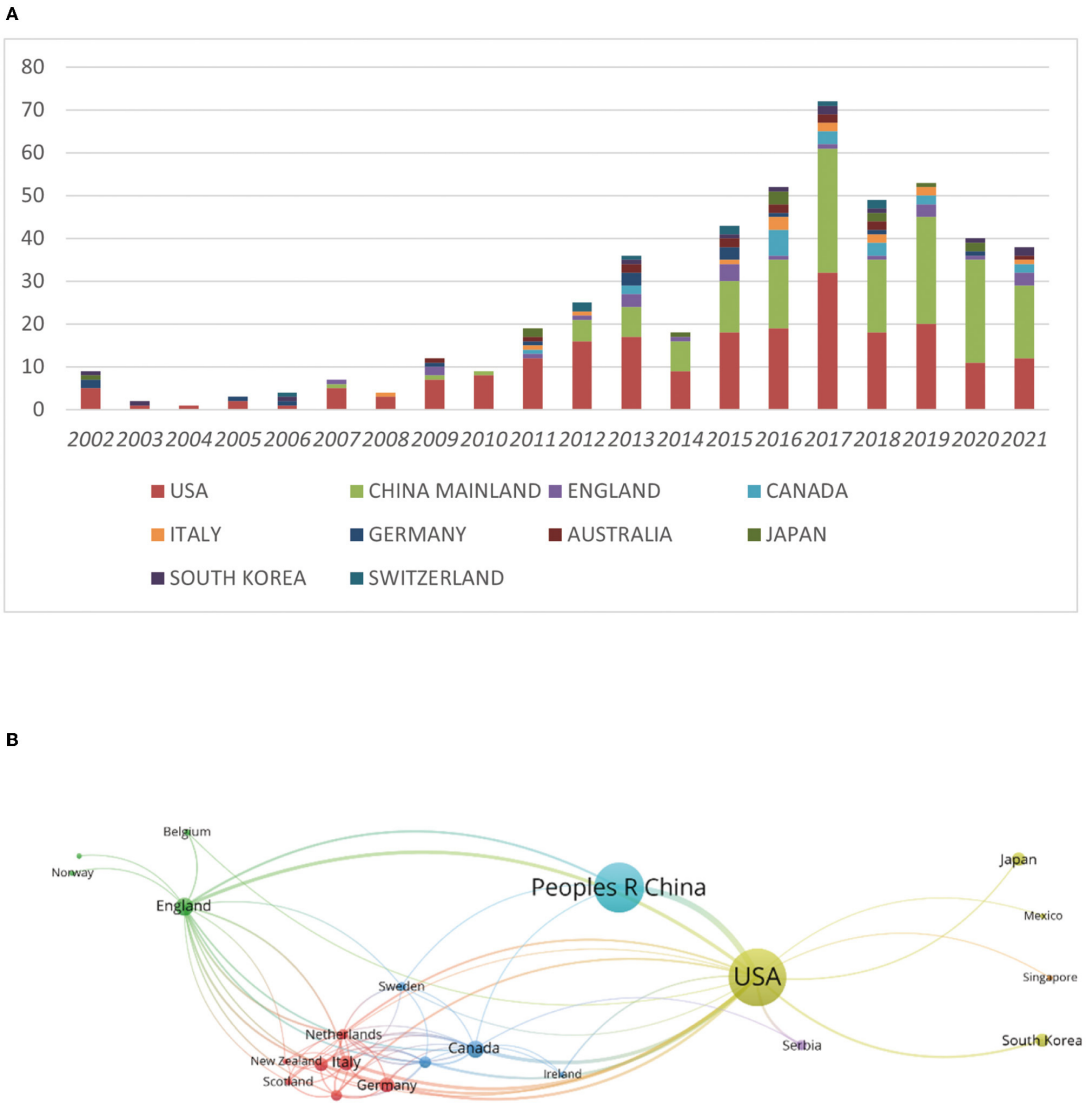
The impact of year and country changes on the number of articles in the field of anesthesia's neurotoxicity to the developing brain. **(A)** Evolution of the number of publications in the 10 most important countries. **(B)** Map of the various countries' close cooperation.

We collected a total of 414 articles by our search criteria. The United States (US) was the country with the highest number of publications in the field of neurotoxicity of anesthesia in the developing brain (*n* = 226), followed by China Mainland (*n* = 167). Except for the above two countries, the number of publications in each of the remaining countries was <25. The US had 10,419 citations, the most of any country, followed by China Mainland (3,064), England (2,009), Italy (1,193), and the Netherlands (1,067). Despite the low number of publications produced, some countries had a high number of mean citations per paper. The Netherlands had the highest mean number of citations per paper (152.43), followed by Scotland (129.75) and Sweden (120.60) (Table 1). Next, we compiled the total number of articles published during this time period by each nation and listed the top 10 nations. These 10 countries published <20 articles per year on the neurotoxicity of anesthesia on the developing brain before 2012. From 2012 to 2017, the number of publications per year gradually increased except in 2014. The number of publications reached a peak in 2017 (Figure 2A). We also analyzed the cooperation between countries for each publication (Figure 2B). In Figure 2B, we could visually see that the USA and China were at the center, and they frequently collaborated with other nations, such as Japan, the UK, and Canada.

**Table 1:** The top 20 countries with the highest number of publications on the neurotoxicity of anesthesia in the developing brain.

**Rank**	**Country**	**Number of papers**	**Total citations**	**Mean citations/Paper**
1	USA	226	10,419	46.10
2	China Mainland	167	3,064	18.35
3	England	23	2,009	87.35
4	Canada	20	1,058	52.90
5	Italy	16	1,193	74.56
6	Germany	15	701	46.73
7	Australia	13	991	76.23
8	Japan	13	432	33.23
9	South Korea	12	411	34.25
10	Switzerland	10	477	47.70
11	Denmark	9	271	30.11
12	Netherlands	7	1,067	152.43
13	Serbia	7	192	27.43
14	Sweden	5	603	120.60
15	France	4	36	9.00
16	Scotland	4	519	129.75
17	Belgium	3	6	2.00
18	Portugal	2	18	9.00
19	Brazil	2	17	8.50
20	Ireland	2	45	22.5

### Authors and institutions

We ranked the top 20 corresponding authors and their institutions according to the number of articles published on anesthetic neurotoxicity in the developing brain (Table 2). There were four professors who had all published eight articles and were cited for first place: Loepke Andreas W from Cincinnati Children's Hospital Medical Center; Wang Cheng from US Food & Drug Administration (FDA); Jiang Hong from Shanghai Jiao Tong University; and Liu Hongtao from China Medical University. The H-index was used to evaluate the academic performance of researchers. The authors with high H-index were Andreas W. Loepke (H-index = 8), followed by Wang Cheng (H-index = 7), Jevtovic-Todorovic Vesna (H-index = 6), and Bai Xiaowen (H-index = 6).

**Table 2:** The top 20 authors with the highest number of publications on the neurotoxicity of anesthesia in the developing brain.

**Name**	**Affiliations**	**Number of papers**	**H-index**
Loepke, Andreas W.	Cincinnati Children's Hospital Medical Center	8	8
Wang, Cheng	US Food and Drug Administration (FDA)	8	7
Jiang, Hong	Shanghai Jiao Tong University	8	5
Liu, Hongtao	China Medical University	8	5
Jevtovic-Todorovic, Vesna	University of Virginia	6	6
Bai, Xiaowen	Medical College of Wisconsin	6	6
Levy, Richard J.	Columbia University	5	4
Zhao, Ping	China Medical University	5	3
Ma, Daqing	Imperial College London	4	4
Zhang, Jun	Fudan University	4	4
Vutskits, Laszlo	University of Geneva	4	4
Sun, Lena S.	Columbia University	4	3
Xie, Zhongcong	Harvard University	4	4
Disma, Nicola	IRCCS Istituto Giannina Gaslini	4	3
Todorovic, Slobodan M.	University of Colorado System	4	3
Davidson, Andrew J.	Royal Children's Hospital Melbourne	3	3
Warner, David O.	Mayo Clinic	3	2
Wei, Huafeng	University of Pennsylvania	3	3
Feng, Xia	Sun Yat Sen University	3	3
Ruzdijic, Sabera	University of Belgrade	3	3

Next, we analyzed the major institutions. The institution with the largest volume of publications was Shanghai Jiao Tong University (*n* = 32), followed by Columbia University (*n* = 31), Harvard University (n = 30), and the League of European Research Universities—LERU (*n* = 29). In the meanwhile, the Royal Children's Hospital Melbourne came in first place for the average number of citations (*n* = 103.67), followed by the US Food and Drug Administration (FDA) (*n* = 101.95), which ranked 19 and six out of 20, respectively.

**Figure 3:**
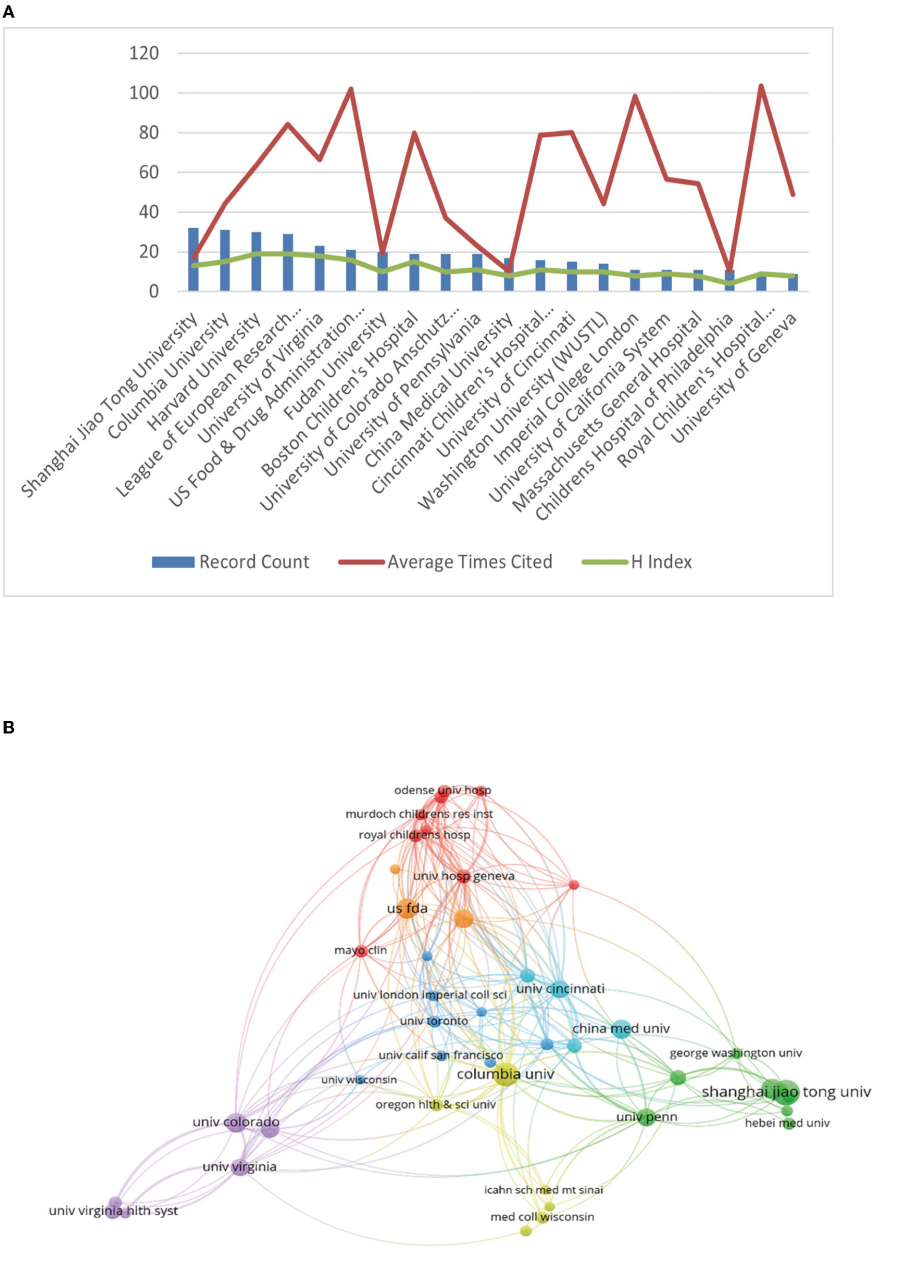
The articles on the neurotoxicity of anesthesia in the developing brain published by different research institutions. **(A)** The top 20 institutions for productivity. The red line shows the typical number of citations per article, the green line shows the H-index of each institution, and the blue bar graph shows the number of articles published by each institution. **(B)** The network visualization map displaying institutional collaborations.

The H-index for each institution was also analyzed. There were two institutions that were ranked highest with an H-index of 19, namely Harvard University and the League of European Research Universities—LERU, followed by the University of Virginia (H-index = 18) and US Food and Drug Administration (H-index = 16). More detailed data are presented in Table 3, Figure 3A. Figure 3B visualizes the close and extensive collaboration between the various institutions with Shanghai Jiao Tong University, US Food and Drug Administration, and Columbia University acting as connecting Bridges.

**Table 3:** The top 20 institutes with the highest number of publications the neurotoxicity of anesthesia in the developing brain.

**Rank**	**Affiliations**	**Number of papers**	**Total citations**	**Mean citations/Paper**	**H-index**
1	Shanghai Jiao Tong University	32	541	16.91	13
2	Columbia University	31	1,371	44.23	15
3	Harvard University	30	1,910	63.67	19
4	League of European Research Universities - LERU	29	2,440	84.14	19
5	University of Virginia	23	1,527	66.39	18
6	US Food & Drug Administration (FDA)	21	2,141	101.95	16
7	Fudan University	20	390	19.50	10
8	Boston Children's Hospital	19	1,518	79.89	15
9	University of Colorado Anschutz Medical Campus	19	702	36.95	10
10	University of Pennsylvania	19	437	23.00	11
11	China Medical University	17	173	10.18	8
12	Cincinnati Children's Hospital Medical Center	16	1,261	78.81	11
13	University of Cincinnati	15	1,200	80.00	10
14	Washington University (WUSTL)	14	619	44.21	10
15	Imperial College London	11	1083	98.45	8
16	University of California System	11	624	56.73	9
17	Massachusetts General Hospital	11	597	54.27	8
18	Childrens Hospital of Philadelphia	11	111	10.09	4
19	Royal Children's Hospital Melbourne	9	933	103.67	9
20	University of Geneva	9	441	49.00	8

### Journal analysis

Next, the journals that publish articles on the neurotoxicity of anesthesia in the developing brain were surveyed, and they were ranked according to the number of publications they had published. Three journals, Anesthesiology, Anesthesia and Analgesia, and Pediatric Anesthesia topped the list with 22 articles published in this period. It is well known that Anesthesiology and Anesthesia and Analgesia are authoritative journals in the field of anesthesia with a Journal Citation Reports (JCR) classification of Q1 and impact factor (IF) of 7.892 and 5.178, respectively. The highest number of relevant papers published in these two journals indicates that research related to the neurotoxicity of anesthesia in the developing brain is very highly regarded and of great interest in the field of anesthesia. The details are shown in Table 4.

**Table 4:** The top 20 journals with the highest number of publications on the neurotoxicity of anesthesia in the developing brain.

**Rank**	**Journal**	**Number of papers**	**Total citations**	**Mean citations/paper**	**Impact factor (IF)**	**Journal citation reports (JCR)**
1	Anesthesiology	22	2,130	96.82	7.892	Q1
2	Anesthesia and Analgesia	22	1,807	82.14	5.178	Q1
3	Pediatric Anesthesia	22	629	28.59	2.556	Q2
4	Journal of Neurosurgical Anesthesiology	19	695	36.58	3.956	Q1
5	Neurotoxicology and Teratology	17	726	42.71	3.763	Q2
6	British Journal of Anaesthesia	16	671	41.94	9.166	Q1
7	Current Opinion in Anesthesiology	12	435	36.25	2.706	Q3
8	Molecular Neurobiology	11	176	16.00	5.59	Q1
9	Neurotoxicity Research	10	356	35.60	3.911	Q2
10	International Journal of Developmental Neuroscience	9	295	32.78	2.457	Q3
11	Plos One	8	151	18.88	3.24	Q2
12	Scientific Reports	8	33	4.13	4.38	Q1
13	Neuroscience	7	125	17.86	3.59	Q3
14	Molecular Medicine Reports	7	86	12.29	2.952	Q3
15	Neurotoxicology	6	98	16.33	4.294	Q2
16	Journal of Anesthesia	6	114	19.00	2.078	Q4
17	International Journal of Molecular Sciences	5	47	9.40	5.924	Q1
18	Toxicological Sciences	5	735	147.00	4.849	Q1
19	Neurochemical Research	5	61	12.20	3.996	Q2
20	Frontiers in Cellular Neuroscience	5	58	11.60	5.505	Q1

**Figure 4:**
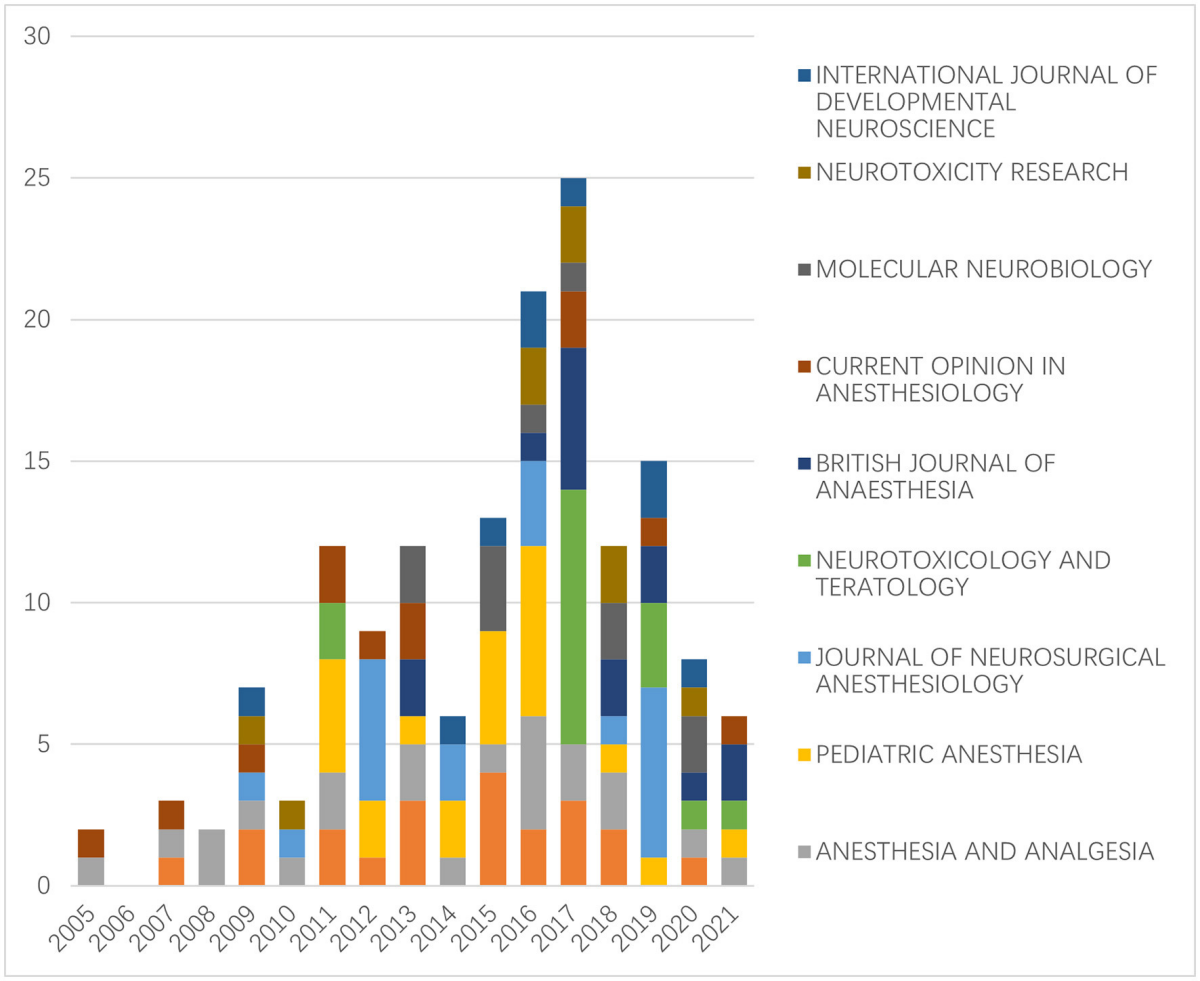
The number of articles on the neurotoxicity of anesthesia in the developing brain published in the top 10 journals each year (no publications in this field in the top 10 journals prior to 2005).

We calculated the annual number of publications in the top 10 journals. No publications in this field were found in the top 10 journals prior to 2005. The highest number of papers were published in the top 10 journals in 2017, and since then, there has been a general decline in that number (Figure 4).

### Subjects and funds

**Figure 5:**
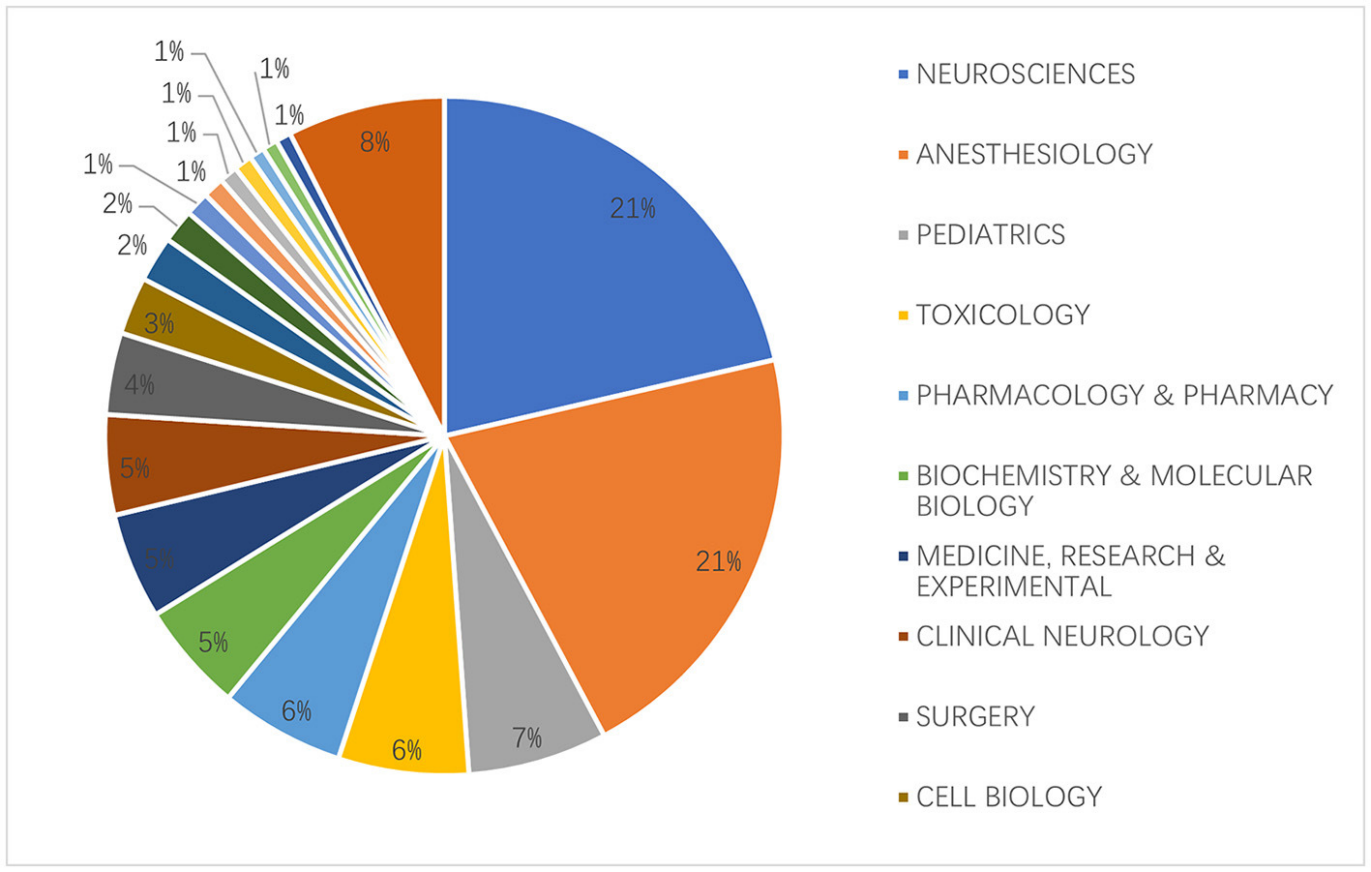
The top 20 subjects related to the neurotoxicity of anesthesia in the developing brain are distributed.

We compiled statistics for each discipline that had articles on anesthetic neurotoxicity in the developing brain after analyzing all disciplines that had such articles. The two disciplines with the largest percentages were, unsurprisingly, neuroscience and anesthesiology, both at 21%, due to our research themes. The above 2 disciplines accounted for nearly half, as shown in Figure 5.

Table 5 lists the top 10 funding institutions, with the first four being the US Department of Health Human Services, National Natural Science Foundation of China, NIH National Institute of Medical Sciences, and NIH Eunice Kennedy Shriver National Institute of Child Health Human Development. The top four funding agencies contributed to 82.5% of the publications, and more than 300 articles were funded. Overall, six of these organizations were headquartered in the US, two in Japan, one in China, and one in Europe.

**Table 5:** The top 10 funding agencies with the highest number of publications on the neurotoxicity of anesthesia in the developing brain.

**Rank**	**Funding agency**	**Number of papers**
1	United States Department of Health Human Services	112
2	National Natural Science Foundation of China NSFC	93
3	NIH National Institute of General Medical Sciences NIGMS	56
4	NIH Eunice Kennedy Shriver National Institute of Child Health Human Development NICHD	50
5	NIH National Institute on Aging Nia	18
6	NIH National Institute of Neurological Disorders Stroke NINDS	13
7	European Commission	11
8	Ministry Of Education Culture Sports Science and Technology Japan MEXT	9
9	Harold Carron Endowment	8
10	Grants In Aid for Scientific Research Kakenhi	7

### The most frequently cited articles

**Figure 6:**
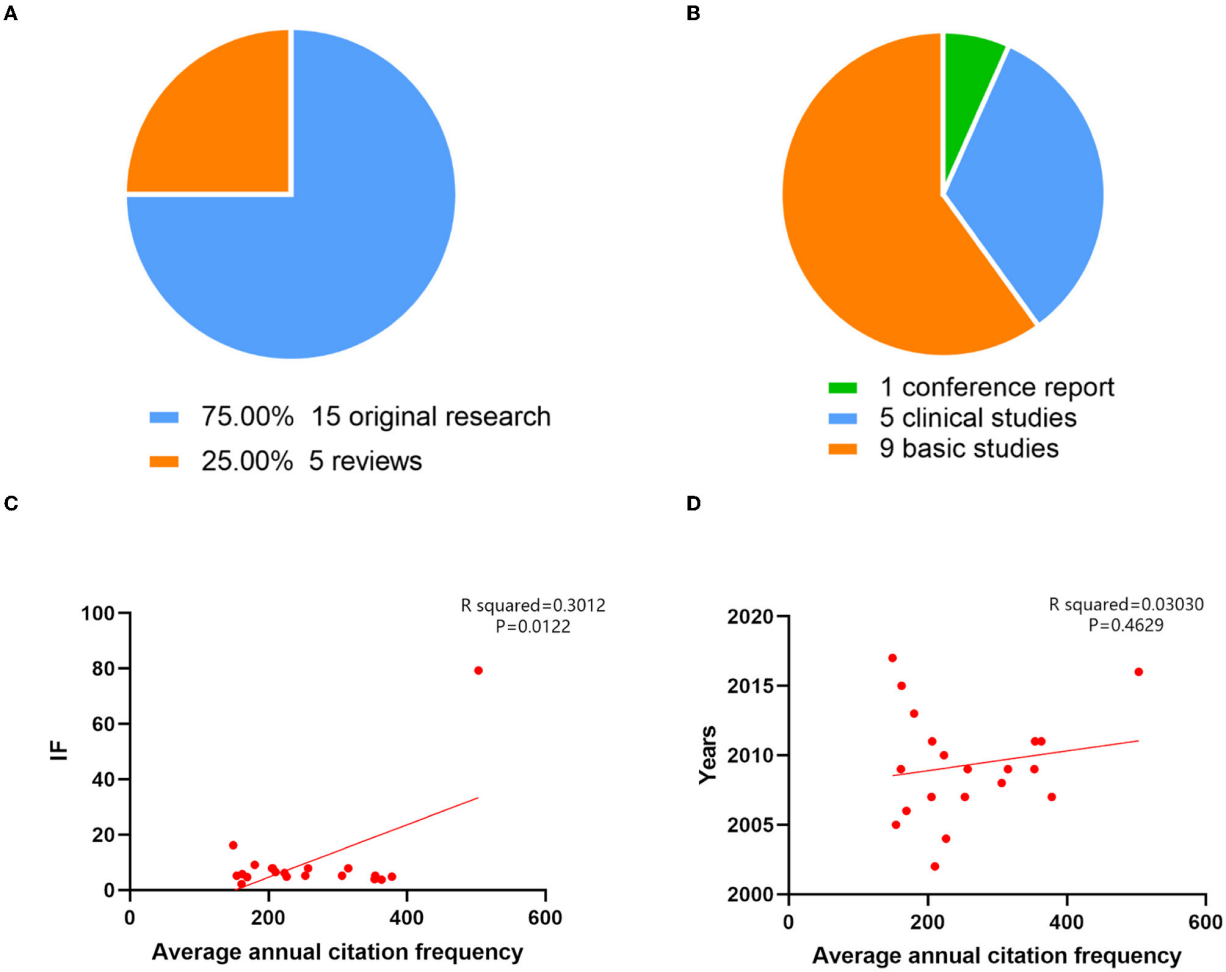
The top 20 cited articles on the neurotoxicity of anesthesia in the developing brain were categorized, and their correlations were examined. **(A)** The article types of the top 20 cited articles on the neurotoxicity of anesthesia in the developing brain. **(B)** The proportion of different article types in original research on the neurotoxicity of anesthesia in the developing brain. **(C)** Based on the correlation analysis, there was a high relationship between the average annual number of citations and the impact factor (R squared = 0.3012; *P* = 0.0122). **(D)** The average annual number of citations and the year of the analysis were unrelated (R squared = 0.03030; *P* = 0.4629).

The top 20 cited publications in the area of neurotoxicity of anesthesia in the developing brain have been listed in Table 6, with quotation counts ranging from 149 to 503. Figures 6A, B make the types of these articles extremely clear. Among these, five were reviews (*n* = 5, 25%), and the remaining 15 articles were original research (*n* = 15, 75%). Original research included one conference report, five clinical studies, and nine basic studies. Four of the five clinical studies were retrospective and only one prospective study, which was also the most cited article, written by Davidson AJ et al., published by Lancet-Journal of the Murdoch Childrens Res Inst in 2016, titled “Neurodevelopmental outcome at 2 years of age after general anesthesia and awake-regional anesthesia in infancy (GAS): an international multicentre, randomized controlled trial.” The final conclusion of this trial was that awake regional anesthesia and sevoflurane anesthesia in infancy are equivalent in terms of neurodevelopmental outcomes at 2 years of age. Other retrospective research expressed extraordinary findings; there was an affiliation between early childhood exposure to anesthesia and an elevated danger of bad neurodevelopmental outcomes. While this association was consistent with preclinical animal data, it may also be explained by confounding effects of surgery, pathology, or basic diseases. Four of the nine basic studies discussed ketamine's characteristics. In conclusion, neonatal rhesus monkeys receiving ketamine for 3 h did not exhibit substantial neurotoxic effects; however, intake of the drug for 9 h or longer, such as for 24 h, was linked to brain cell death. The remaining five fundamental studies all mentioned isoflurane. Four investigations showed that exposure to 0.75% isoflurane for 6 h, either alone or in combination with other gases, induced neurotoxicity. Another study impaired memory by providing 1.7% isoflurane for 35 min each day for 4 days.

**Table 6:** The top 20 highest-cited articles on the neurotoxicity of anesthesia in the developing brain.

**Rank**	**Title**	**Corresponding author**	**Affiliation**	**Source title**	**Year of publication**	**Cited by**
**1**	Neurodevelopmental outcome at 2 years of age after general anaesthesia and awake-regional anaesthesia in infancy (GAS): an international multicentre, randomised controlled trial	Davidson, AJ	Murdoch Childrens Res Inst	Lancet	2016	503
**2**	Ketamine-induced neuronal cell death in the perinatal rhesus monkey	Slikker, W	US FDA	Toxicological Sciences	2007	378
**3**	Ketamine anesthesia during the first week of life can cause long-lasting cognitive deficits in rhesus monkeys	Paule, MG	US FDA	Neurotoxicology and Teratology	2011	363
**4**	Early Childhood Exposure to Anesthesia and Risk of Developmental and Behavioral Disorders in a Sibling Birth Cohort	DiMaggio, C	Columbia Univ	Anesthesia and Analgesia	2011	354
**5**	A Retrospective Cohort Study of the Association of Anesthesia and Hernia Repair Surgery with Behavioral and Developmental Disorders in Young Children	DiMaggio, C	Columbia Univ	Journal of Neurosurgical Anesthesiology	2009	353
**6**	Dexmedetomidine Attenuates Isoflurane-induced Neurocognitive Impairment in Neonatal Rats	Maze, M	Univ London Imperial Coll Sci Technol & Med	Anesthesiology	2009	315
**7**	An assessment of the effects of general anesthetics on developing brain structure and neurocognitive function	Loepke, AW	Cincinnati Childrens Hosp	Anesthesia and Analgesia	2008	306
**8**	Behavior and Development in Children and Age at the Time of First Anesthetic Exposure	Kalkman, CJ	Univ Med Ctr Utrecht	Anesthesiology	2009	257
**9**	Use of anesthetic agents in neonates and young children	Rappaport, BA	US FDA	Anesthesia and Analgesia	2007	253
**10**	Developmental neurotoxicity of ketamine: Morphometric confirmation, exposure parameters, and multiple fluorescent labeling of apoptotic neurons	Scallet, AC	US FDA	Toxicological Sciences	2004	226
**11**	Isoflurane anesthesia induced persistent, progressive memory impairment, caused a loss of neural stem cells, and reduced neurogenesis in young, but not adult, rodents	Zhu, CL	Univ Gothenburg	Journal of Cerebral Blood Flow and Metabolism	2010	223
**12**	Drug-induced apoptotic neurodegeneration in the developing brain	Olney, JW	Washington Univ	Brain Pathology	2002	210
**13**	Comparison of the Neuroapoptotic Properties of Equipotent Anesthetic Concentrations of Desflurane, Isoflurane, or Sevoflurane in Neonatal Mice	Loepke, AW	Cincinnati Childrens Hosp	Anesthesiology	2011	206
**14**	Xenon mitigates isoflurane-induced neuronal apoptosis in the developing rodent brain	Ma, DQ	Univ London Imperial Coll Sci Technol & Med	Anesthesiology	2007	205
**15**	Anaesthetic neurotoxicity and neuroplasticity: an expert group report and statement based on the BJA Salzburg Seminar	Jevtovic-Todorovic, V	Univ Virginia	British Journal of Anaesthesia	2013	180
**16**	General anesthesia activates BDNF-dependent neuroapoptosis in the developing rat brain	Jevtovic-Todorovic, V	Univ Virginia Hlth Syst	Apoptosis	2006	169
**17**	Propofol: A Review of its Role in Pediatric Anesthesia and Sedation	Chidambaran, V	Cincinnati Childrens Hosp Med Ctr	CNS Drugs	2015	162
**18**	Prolonged exposure to ketamine increases neurodegeneration in the developing monkey brain	Wang, C	US FDA	International Journal of Developmental Neuroscience	2009	161
**19**	Revising a dogma: Ketamine for patients with neurological injury?	Himmelseher, S	Univ Virginia	Anesthesia and Analgesia	2005	154
**20**	Association of Anesthesia and Surgery During Childhood with Long-term Academic Performance	Glatz, P	Kalmar Cty Hosp	JAMA Pediatrics	2017	149

Additionally, we examined the relationship between the average number of citations, year of publication, and impact factors of the 20 most cited journals. There was no connection between the average number of citations and the year of publication (R2= 0.03030, *P* = 0.4629). The mean number of citations and the impact factor did, however, significantly correlate with one another (R2 = 0.3012, *P* = 0.0122) (Figures 6C, D).

### Research hotspots and publication trends

**Figure 7:**
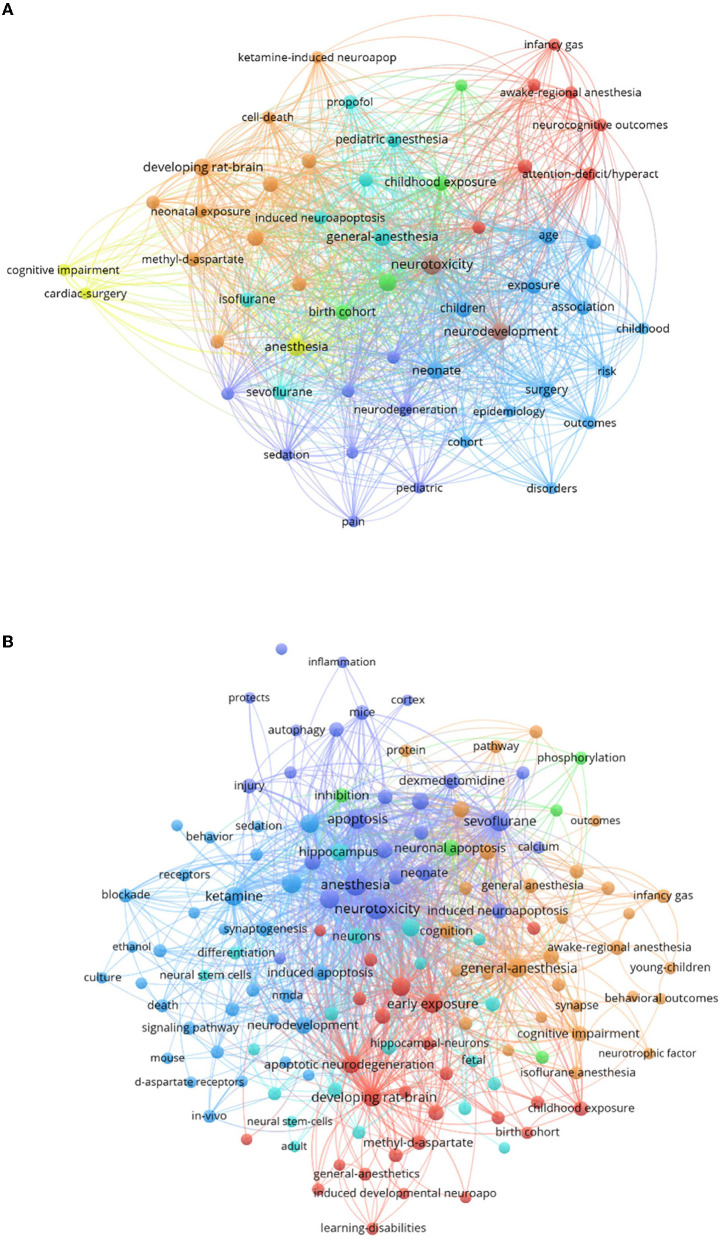
A summary of the hotspot trends of articles on the neurotoxicity of anesthesia in the developing brain. The diameter of the circles and the thickness of the lines show how frequently certain keywords occur together. **(A)** Clinical research hotspots of articles published on the neurotoxicity of anesthesia in the developing brain. **(B)** Basic research hotspots of articles published on the neurotoxicity of anesthesia in the developing brain.

Keywords are the core of an article and reflect the topic of the article; by analyzing the keywords, you can learn about the current hotspots being researched in the field. We generated a keyword co-occurrence network through CiteSpace, in which the larger the node, the more frequently the node co-occurs with the central node, which additionally suggests a nearer relationship between the two. We anticipated that the research hotspots on anesthetic neurotoxicity in the developing brain would differ depending on clinical investigations and basic research; therefore, we analyzed them individually. In Figure 7A, we learned that clinical studies were more focused on the end result: “cognition,” “attention,” “neurocognitive outcomes,” “attention-deficit/hyperact,” and “cognitive impairment” were the keywords that appear more frequently. The keywords for basic research were more complex relatively: “synaptogenesis,” “apoptosis,” “signaling pathway,” “NMDA,” “inflammation,” and “autophagy” appeared more frequently in Figure 7B, indicating that the basic trials focused mainly on the exploration of mechanisms.

## Discussion

The neurotoxicity consequences of anesthetic exposure on the developing brain have been one of the current research hotspots and numerous articles were published in the past decades. Several preclinical studies on the neurotoxicity of anesthesia indicated that exposure to general anesthesia may lead to structural and functional abnormalities in the brain (12). Most general anesthetics have these adverse effects, particularly ketamine and isoflurane, as reflected in this article. As a result, serious questions about the security of young children undergoing frequent or prolonged anesthesia have been raised and the US Food and Drug Administration has released a safety notification in this regard (13, 14). On the applicability of the findings of basic research trials to humans, however, expert opinion is still divided. As anesthesia may adversely affect millions of children requiring surgery every year, more research is required to fully understand the dangers of anesthetic neurotoxicity. Here, 414 articles were solicited from the Science Citation Index extended database, and through a bibliometric analysis, we gave a brief overview of developments in the study of the neurotoxicity of anesthesia on the developing brain.

Overall, research in the area of neurotoxicity of anesthesia to the developing brain culminated in 2017, with a decreasing trend thereafter. This may be due to the fact that as research into anesthetics continues, drugs that are clearly neurotoxic are no longer clinically used in anesthesia and are, therefore, no longer studied in this area.

The US has the most publications and citations among these nations, with China ranking second after the US. The United States has become recognized as an authority in the field thanks to the study of experts such as Andreas W. Loepke, Wang Cheng, Jevtovic-Todorovic Vesna, Bai Xiaowen, and Richard J. Levy. Andreas W. Loepke appears to have undergone the most extensive research, as evidenced by the highest H-index. The position of China is mainly supported by experts, such as Jiang Hong, Liu Hongtao, Zhao Ping, and Zhang Jun.

In terms of journals, most of the articles in the field of neurotoxicity of anesthesia on brain development are published in leading journals in the field of anesthesia, such as Anesthesiology and Anesthesia and Analgesia. This shows that the topic we are studying is of great importance in the field of anesthesia. The results of the hotspot analysis probably reflect the fact that clinical studies have focused on whether early exposure to anesthesia affects later cognition, behavior, and attention, whereas basic research has focused more on how anesthesia causes neurotoxicity. There are currently no uniform findings on any of these. We discovered through statistical analysis that the fields of neuroscience and anesthesiology publish more articles on the neurotoxicity of anesthesia in the developing brain. It could be the cause that the majority of fundamental studies are published in journals connected to neuroscience, while many clinical studies are published in journals linked to anesthesia.

A large number of clinical and preclinical studies have been motivated by the FDA's warnings. Conducting prospective, randomized studies on human infants and children without confounding factors is challenging due to ethical and technical issues. Three clinical investigations are currently more well-known than the others. One of these is the General and Spinal Anesthesia (GAS) study, which is the study conducted by the most cited article of the last 20 years mentioned in this article. The study concluded that there was no proof that early single exposure of kiddies to sevoflurane for <1 h elevated the hazard of altered neurodevelopmental consequences at 2 years of age in contrast with awake regional anesthesia (15). The GAS study reported in 2019 increased the age of the study to 5 years and still found no neurodevelopmental alterations (16). Another trial called the Pediatric Anesthesia and Neurodevelopmental Assessment (PANDA) similarly concluded that young children who received one anesthesia before 36 months were not significantly different from those who did not receive anesthesia in terms of intelligence (4). The primary outcome of the significant study called Mayo Anesthesia Safety in Kids (MASK) also showed that exposure to general anesthesia before the age of 3 years was not associated with significant differences in general cognitive ability as quantified by Full Scale IQ score, relative to unexposed children. However, the secondary outcomes of this study showed that multiple, but not single, exposures were associated with modest decreases in processing speed and fine motor coordination (17). Our focus has changed since the MASK study from “single exposure” to “multiple exposure,” which is a significant advancement. These three studies demonstrate that there is no proof in favor of the hypothesis that one or more anesthetic exposures during early infancy lower intelligence quotient levels in adulthood. However, a recent national population-based cohort study, comparing 11,457 children under 2 years of age who received general anesthesia with 22,914 children who did not, showed that receiving anesthesia early (before 2 years of age) may increase developmental delay; both increased anesthesia frequency and longer overall anesthesia duration increased this risk (18). It also reminds us that not only the type of anesthesia but also the frequency and duration of anesthesia are crucial. Recent clinical research outcomes are still contradictory.

Preclinical studies were a major player in general anesthetic toxicity studies, which have mostly been conducted on rodent models. There is uncertainty as to whether the mechanisms observed in rodent models are clinically relevant (19). Rodents are genetically more distant from humans than non-human primates (NHPs) (20). However, studies using NHPs are limited due to their small numbers and high cost. From the analysis for basic study in this field, we could observe apoptosis, calcium, phosphorylation, synaptogenesis, neurotrophic factor, NMDA, d-aspartate receptors, methyl-d-aspartate, and neural stem cells were research hotspots from 2002 to 2021. Currently, commonly used anesthetics are thought to act through NMDA and GABA receptors, but the specific mechanisms by which anesthesia causes neurodevelopmental disorders are highly complex (21). The keywords that appear in the analysis of basic research hotspots are neuroinflammation, apoptosis, synaptogenesis, and autophagy, all of which are mechanisms of anesthesia-induced neurotoxicity. Many studies have explored specific signaling pathways. The mechanism most frequently cited when discussing anesthesia-induced neurodevelopmental disorders is neuroinflammation. Microglia are the main mediators of neuroinflammation in the brain, and they can polarize into M1 types, which are harmful to neurons, and M2 types, which contribute to tissue repair (22). Sevoflurane may enhance M1 polarization while inhibiting M2 activation (23). It has been suggested that sevoflurane and isoflurane may increase interleukin-6 (IL-6) levels through the nuclear factor-κB (NF-κB) signaling pathway, thereby inducing neuroinflammation and cognitive impairment, and that inhibition of the NF-κB signaling pathway may rescue sevoflurane-induced inflammation and reduce cognitive dysfunction in adulthood (24, 25). Apoptosis is the active, orderly death of cells under certain physiological or pathological conditions, controlled by genes (26). It has been demonstrated that general anesthesia may cause widespread apoptotic neuronal death in the developing brain of baby mice, rats, and rhesus monkeys (27, 28). Early exposure to general anesthetics may impair neuronal plasticity, according to several animal studies (29–33). Autophagy is a double-edged sword, with some studies showing that autophagy causes neurotoxicity, while others confirm its neuroprotective effects. The specific effects of autophagy need to be further investigated (34). Other mechanisms include oxidative stress and iron death (34), which are not represented in the basic research hotspot analysis map, probably due to their low number of studies at present. For specific signaling pathways, Jiaojiao Wang et al. have reviewed the current signaling pathways for postoperative neurotoxicity in neonates due to general anesthesia, such as HIPK2/Akt/mTOR, PI3K/Akt, and JAK/STAT, with a view to providing an important reference for the treatment of clinical disease (35).

In particular, the large circle for “sevoflurane” in the base hotspot clustering diagram indicates that there have been a lot of preclinical studies carried out on it. This may be due to the low solubility of sevoflurane in blood gas and its relatively rapid onset of action and the fact that it is currently the most commonly used inhalation anesthetic in clinical pediatrics and researchers are anxious to know its safety. Repeated or prolonged exposure to sevoflurane has been shown in several animal studies to cause neurotoxicity in the developing brain (28, 36, 37), but the results of clinical studies are not always consistent. As with other anesthetics, the potential mechanisms by which sevoflurane causes neurotoxicity are controversial. A recent review specifically summarized its possible mechanisms, including nerve cell damage and death, tau protein phosphorylation, and neuroendocrine abnormalities (38).

On the one hand, it is known from current studies that the probability of a single, short exposure to anesthesia causing neurotoxicity is particularly small, and future clinical research will be interested in the dose, duration, and frequency of anesthetic exposure. On the other hand, animal experiments are relatively easy to carry out, but it will be worth thinking about how to select animal models, whether the mechanisms observed in animal models are clinically relevant, and how to translate preclinical results into clinical practice.

Our bibliometric analysis also had some limitations. First, our search was conducted in June 2022 and relevant articles published after that date were not included in this study. Second, the earlier the article was published, the more citations it received. The “top 20 most cited articles” did not include all authoritative articles that have been published more recently. This is a result of insufficient citations. Although the correlation between the years of publication and the number of citations of the top 20 articles shows that there is no correlation between the two, time is still a factor to be considered. Finally, there is no doubt that journals with high-impact factors are more favored and that articles published in them are more likely to be cited by other articles, suggesting that high-impact factors inherently lead to bias.

## Conclusion

We looked through and evaluated 414 English-language articles on the subject of the neurotoxicity of anesthesia in the developing brain. Although our study has some limitations, it is still meaningful. Our study found that research in this area peaked in 2017. Various countries, led by the United States and China, are exploring and researching this area. Current clinical studies are mainly retrospective, and in the future, we should place more emphasis on prospective, multicenter, long-term follow-up clinical studies. More basic research is also needed on the neurotoxic mechanisms of anesthesia in the developing brain.

## Data availability statement

The original contributions presented in the study are included in the article/supplementary material, further inquiries can be directed to the corresponding authors.

## Author contributions

TL and HM conceived and designed the structure of this manuscript and revised the manuscript. YC, YS, KY, XL, DG, and JY analyzed and wrote the manuscript. All authors contributed to the article and approved the submitted version.
